# mRNA microarray profiling identifies a novel circulating HTRA2 for detection of gastric cancer

**DOI:** 10.1002/jcla.24054

**Published:** 2021-10-28

**Authors:** Liangliang Wu, Xiao Li, Xin Chen, Fan Wu, Guangshun Sun, Ye Cheng, Weiwei Tang, Wenling Zhang, Chengyu Lv

**Affiliations:** ^1^ Department of General Surgery Nanjing First Hospital Nanjing Medical University Nanjing China; ^2^ Hepatobiliary/Liver Transplantation Center Key Laboratory of Living Donor Transplantation The First Affiliated Hospital of Nanjing Medical University Chinese Academy of Medical Sciences Nanjing China; ^3^ Department of Gastroenterology The First Affiliated Hospital of Nanjing Medical University Nanjing China

**Keywords:** diagnosis, gastric cancer, HTRA2, immune, microarray

## Abstract

**Background:**

mRNAs have been shown to be critical biomarkers or therapeutic targets for human diseases. However, only a few of them have been studied as blood‐based biomarkers for gastric carcinoma (GC) detection.

**Methods:**

mRNA expression profiles for GC were screened using plasma samples from 10 GC patients with different TNM stages and 5 healthy individuals as controls. One candidate tumor‐related mRNA named HTRA2 was then evaluated in GC samples with quantitative real‐time polymerase chain reaction (qRT‐PCR). TCGAportal, UALCAN, and TISCH database were used to explore the function of HTRA2 in GC. Finally, the effect generated by HTRA2 expression on cell proliferating, invading, and migrating processes was assessed in vitro with knockdown and over‐expression strategies.

**Results:**

HTRA2 displayed noticeable increase inside GC plasma compared with control cases. Higher expression of HTRA2 displayed a correlation to higher clinicopathological stage and worse prognosis. HTRA2 knocking down down‐regulated GC cells' proliferating, invading, and migrating states, while HTRA2 over‐expression exerted the inconsistent influence. HTRA2 protein, which may interact with PINK1, PARL, and CYCS, was mainly located in the mitochondria of cells and primarily involved cellular response and metabolic signaling pathway. Immune factors may interact with HTRA2 in GC, and HTRA2 was found noticeably linked with immunosuppressor such as CD274, IDO1, and TIGIT.

**Conclusion:**

One plasma HTRA2 can be an emerging diagnosis‐related biomarker to achieve GC detecting process, but the particular regulatory effect still needs to be further explored.

## Graphical abstract

mRNA expressing state overviews received the screening process inside GC, with samples of plasma of 10 GC cases and 5 normal controls. High‐throughput sequencing screened HTRA2 positively correlated with gastric cancer malignant cell phenotype. HTRA2 was then quantitated and evaluated with qRT‐PCR. As revealed from the results, HTRA2 displayed noticeable increase inside GC plasma in contrast with control cases. According to experiment‐related procedures in vitro, HTRA2 knocking down down‐regulated GC cells' proliferating, invading, and migrating states, while HTRA2 over‐expression exerted the inconsistent influence. To confirm HTRA2 was derived from GC tumor, we further detected in primary cancerous and neighboring noncancerous tissues from GC cases. Results showed HTRA2 expressing states were significantly higher than that of neighboring noncancerous tissues. In conclusion, HTRA2 promotes malignant phenotype of gastric cancer.

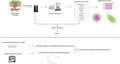

## 1 INTRODUCTION

Gastric carcinoma (GC) is the fifth most common carcinoma worldwide and the 3rd major factor causing carcinoma‐associated death globally.^1^ Particularly, the incidence ratio of GC has been increasingly risen in Eastern Asia. It will conduct the metastasizing in nearby tissues and organs via lymph nodes and the generation of more carcinoma cells via blood^2^ that metastasis and recurrence are appearing under large ratios.^3^, ^4^ Nowadays, endoscopically related and pathologic examining processes refer to the normal tools in terms of carcinoma diagnosing process. Regardless of their sensitive state and particular property for visualizing and locating malignant locations, the mentioned methods are invading essentially, impeding cases based on screening procedure in terms of gastric carcinoma, which leads to numerous cases diagnosed with advanced GC at the first diagnosis time. For this reason, powerful biomarkers and their molecular systems should be explored for clarifying the gastric malignancies' pathophysiological knowledge.

Messenger RNA (mRNA) is progressively critical to clinically related uses (eg, disease diagnosing process and carcinoma cases' prognosis) in the past few years. mRNAs expressing profiling processes with microarray are capable of differentiating common from carcinoma tissues and of classifying various tumors and relevant grades. In addition, particular mRNAs expressing characteristics were reported with a correlation to carcinoma prognostic process, so it may be capable of determining treating course. HTRA2 refers to one mitochondria protein exerting the dual effect on cell physiological knowledge. The mentioned protease keeps mitochondria homeostasis facilitating cell surviving state. Besides, HTRA2 is likely to cause irreversibly damaged cells to die through the promotion of cell death. Additionally, HTRA2 expressing state decreases inside several tumors and increases inside other tumors,^5^, ^6^, ^7^ demonstrating that HTRA2 expressing state changes following tumor category. Currently, information regarding circulating HTRA2's implication inside GC is rare, and the expressing state exhibited by the HTRA2 in GC and the relevant correlations to clinicopathology‐related characteristics are not clear.

Here, mRNA expression profiles for GC were screened using plasma samples covering 5 control groups, 5 cases with T3N1‐3M0, and 5 cases with T3N0M0. As indicated from disease enrichment term, KEGG channel, and gene ontology (GO) analyses, numerous mRNAs had implication implied during carcinogenesis. Candidate tumor‐related HTRA2 mRNA was then quantitated in GC tissues and plasmas, respectively. The high expression of HTRA2 mRNA positively contributes to cell proliferating, invading, and migrating processes. Therefore, HTRA2 may serve a novel circulating biomarker for detection of GC.

## 2 METHODS

### 2.1 mRNA microarray

CapitalBio Technology Human mRNA Array had 4 completely the same arrays per slide. Overall RNA received the extraction according to plasmas with the TRIzol reagent (Invitrogen) as well as the purification based on the use of mirVana miRNA Isolation Tool (Ambion). cDNA under the labeling based on one fluorescent dye received the production with Eberwine's linear RNA amplifying approach. The mRNA array information received the analysis in terms of information summarizing process, normalizing process, and quality controlling process with the GeneSpring software V13.0 (Agilent).

### 2.2 Cases and samples

We acquired peripheral blood (5 ml) of GC cases prior to the operating process. Next, the plasma received the isolation. By complying with the age‐and‐gender matching standard, fresh plasma samples which were not abnormal received the collection according to patients in Nanjing First Hospital from 2018 to 2020. In terms of overall peripheral blood samples, the anti‐coagulant was ethylenediaminetetraacetic acid (EDTA). We acquired GC tissues and relevant samples according to cases carrying GC, having undergone surgery from 2018 to 2020 in Nanjing First Hospital. Prior to the operating process, the cases underwent no radiotherapeutic or chemotherapeutic processes. The mentioned tissue specimens received the immediate storing under −80°C based on one refrigerating element till using. Based on the standard, the paired neighboring non‐tumor tissues had the confirmation to cover not any cells of tumor by pathologically related analyzing process and had the location of 5 cm from the GC edge. The present work gained the approval from the Ethics Committee of Nanjing First Hospital, Nanjing Medical University. Prior to their engagement, all cases presented informed consent in a written form.

### 2.3 RNA isolation, reverse transcription, and quantitative real‐time polymerase chain reaction (qRT‐PCR)

Overall RNA according to tissues under pairing received the extraction with TRIzol reagent (Thermo Fisher Scientific) and overall RNA inside plasma received the extraction with the use of TIANamp Virus RNA Tool by complying with the guidelines of the producer. Based on the Prime‐Script™ RT‐PCR tool (Takara) by complying with the guidelines of the producer, we acquired overall cDNAs. HTRA2 expressing state was determined by qRT‐PCR with the primer pair, that is, 5′‐ CTCCCCGGAGTCAGTACAACT‐3′ (Forward, or F) and 5′‐ AGGATCTCGATATAGACCACGG‐3′ (reverse, R). Glyceraldehyde 3‐phosphate dehydrogenase (GAPDH) became one internal control.

### 2.4 Co‐expression analysis

Co‐expression analysis is based on correlations in mathematics and to find mRNA‐non‐coding RNA pairs whose expression profiles are similar from the gene expressing state. A large number of functionally relevant genes exhibit consistent expressing profiles based on related conditions, in particular genes under the common regulation using normal transcribing‐related elements or genes whose product constitutes the same protein complex or genes which participate in the same regulatory path. Therefore, co‐expression analysis and network construction based on co‐expression results can help users to discover the possible relationship between mRNA and non‐coding RNAs and can find mRNAs that affect the regulation of non‐coding RNA expression.

### 2.5 Cell culturing and transfecting processes

SGC‐7901 and MGC‐803 cells received the culturing process using RPMI 1640 medium (BI), involving 10% fetal bovine serum (FBS) (GiGCo) under 37℃ inside one 5% CO_2_ chamber covering streptomycin (100 mg/ml) and penicillin (100 IU/ml). Non‐targeting control siRNA (si‐NC) and small interfering RNA against HTRA2 (si‐HTRA2) were produced by Hongxin Biological Technology Co., Ltd. The study acquired cells for exploring the information to carry out 48 h post‐transfecting process. HTRA2 (human) siRNA: 5′‐ UCGCAGAUGUGGUGGAGAATT‐3′.

### 2.6 Plasmids constructing and transfecting process

Hongxin Biological Technology Co., Ltd. (China) offered the control plasmid vector pcDNA3, as well as the HTRA2 over‐expression plasmid pcDNA3‐HTRA2. Plasmid pcDNA3‐HTRA2 over‐expressed, using pcDNA3.1 (+) to be vector. 1 × 10^6^ cells received the seeding process inside 6‐well plates and the 24 h culturing process. After 12–16 h, serum‐free medium received the switch as a complete medium supplemented by 10% FBS. The cells under the transfection can be employed to conduct the in‐depth experimental processes. The transfecting efficiency reached over 85%.

### 2.7 Cell proliferation experiments

During the clonogenesis experiment, transfected cells were inoculated in 6‐well plates at a density of 1000 cells per well and then cultured in RPMI 1640 medium containing 10% FBS. After 10 days, the cells were imfixed based on the use of methanol and then stained with GIMSA. Finally, the colony is imaged and counted. In the ELL Counting Kit‐8 (CCK‐8) test process, MGC‐803 and SGC‐7901 cells were transfected and incubated at 37°C first. Next, ccK‐8 solution (Biosharp) was introduced and incubated for 2 h in their respective wells. The absorbance received the measuring process at 0, 24, 48, 72, and 96 h at 450 nm.

### 2.8 Transwell migration and invasion assays

SGC‐7901 and MGC‐803 cells were inoculated with 200 μ L serum‐free RPMI 1640 medium in the upper compartment. The Transwell chamber (Corning) received the paving process using a matrix glue mixture (BD Biosciences) for the invasion test process and did not use the matrix glue mixture for the migration test. RPMI 1640 medium and 10% FBS were introduced into the bottom chamber to act as chemical attractants for GC cells. When the 24‐h culture process was complete, the upper chamber was immobilized and then stained for 15 min based on crystal violet (Kagan).

### 2.9 Wound healing assay

MGC‐803 and SGC‐7901 cells were transfected on 6‐well culture plates. Using a standard 20 μl pipette tip, the artificial linear wound was eliminated on the fused cell monolayer. Free‐floating cells and debris isolated from the bottom of the well are slowly removed. Medium was introduced, and the plate was incubated at 37℃. Scratch widths were recorded under an inverted microscope and photographed at 0, 24, and 48 h.

### 2.10 HTRA2 expression level analysis and clinicopathological analysis

TCGAportal was used to investigate the expression of HTRA2 in different tumor tissues and corresponding para‐carcinoma tissues. The Human Protein Atlas database was used to examine HTRA2 expression in different tissues. UALCAN refers to a comprehensive and interactive web resource to delve into cancer OMICS data, which was used here to compare HTRA2 expression in GC patients of different races, ages, and molecular subtypes. Tumor Immune Single‐cell Hub (TISCH) is a single‐cell RNA‐sequencing (scRNA‐seq) database focusing on tumor microenvironment. Kaplan‐Meier Plotter was used to compare correlations between HTRA2 expression and overall survival (OS), first progression (FP), and progression‐free survival (PFS).

### 2.11 Tools for HTRA2 location in cells and enrichment analysis of HTRA2

The Human Protein Atlas was utilized for obtaining HTRA2 location in cells. We used Metascape and STRING to create a network of interactions between HTRA2 and other important proteins and pathways. TISIDB, a web portal for tumor and immune system interactions, integrating a range of heterogeneous data, was adopted for delving into the spearman correlations between HTRA2 and immune‐modulator expression.

### 2.12 Statistics‐related analyzing process

The continuing information received the comparative analysis by performing one individual t‐testing process of the 2 groups. Statistics‐related analyzing process was performed with SPSS (Version 22.0) and presented graphically in GraphPad Prism 8.0. A *p* value of 0.05 was considered to be statistically significant.

## 3 RESULTS

### 3.1 Genome‐wide profiles were produced for patient/control plasma samples to identify candidate mRNAs

**FIGURE 1:**
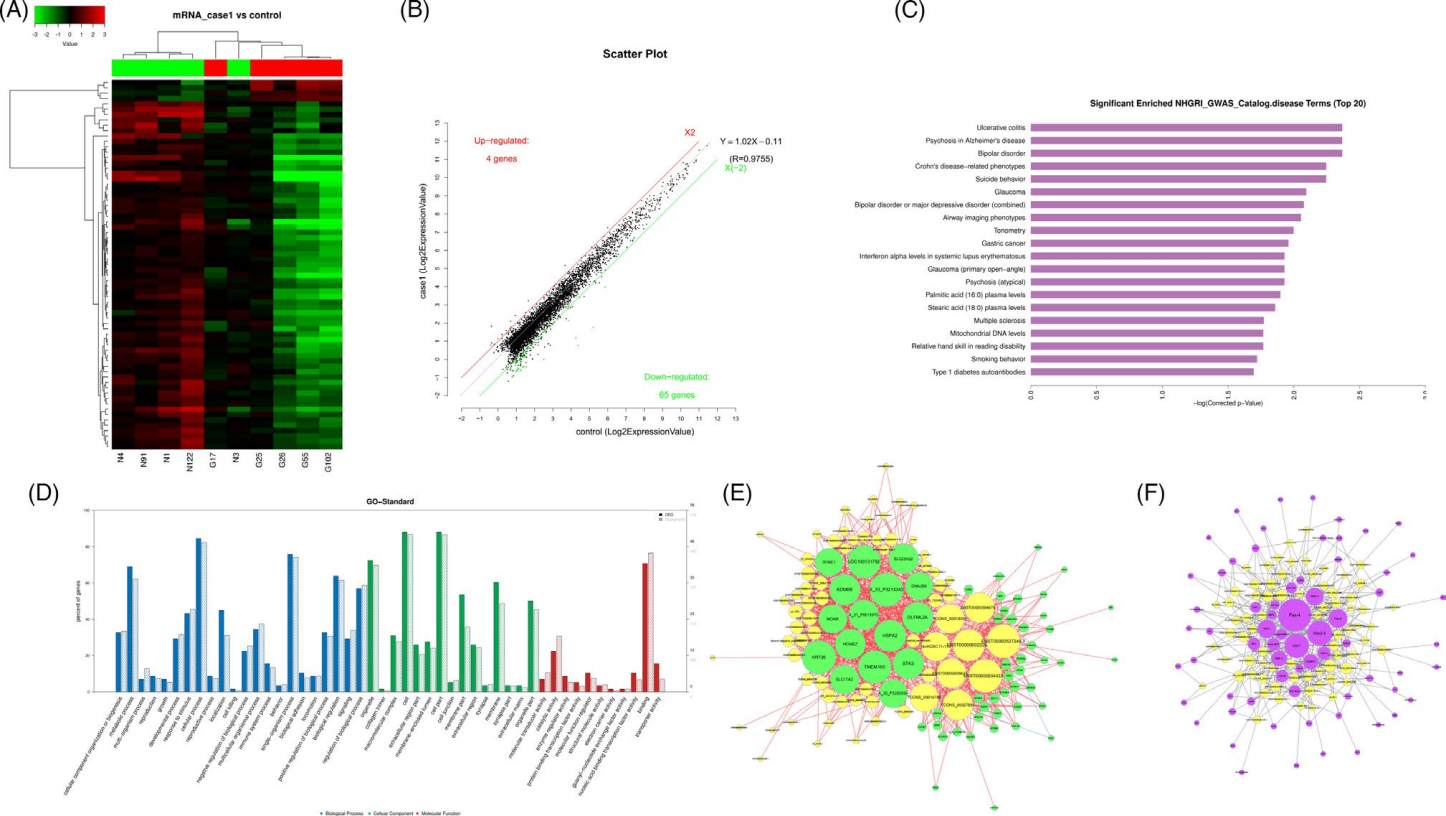
Profiling of mRNAs in the plasmas from gastric carcinoma (GC) patients with early TNM stages and normal controls. (A) Heat map shows the up‐regulated and down‐regulated mRNAs in case 1 vs. control group. (G for GC, and N for normal individuals' plasma). Each column represents the expression profile of a tissue sample, and each row corresponds to a mRNA. High expression level is indicated by “red” and lower levels by “green.” (B) Scatter plot shows the up‐regulated and down‐regulated mRNAs in case 1 vs. control group. Higher expression levels are indicated by “red,” lower expression levels are indicated by “green,” and no significant difference is indicated by “black”. (C) Disease pathway analysis of mRNAs in case 1 vs. control group. (D) GO analysis of mRNAs in case 1 vs. control group. (E) Co‐expression analysis and cytoscape software were used to perform the two‐by‐two correlation calculation and hypothesis verification of mRNAs and non‐coding RNAs. (F) Cis‐prediction was performed to predict the association between mRNA and non‐coding RNAs according to their locations

**FIGURE 2:**
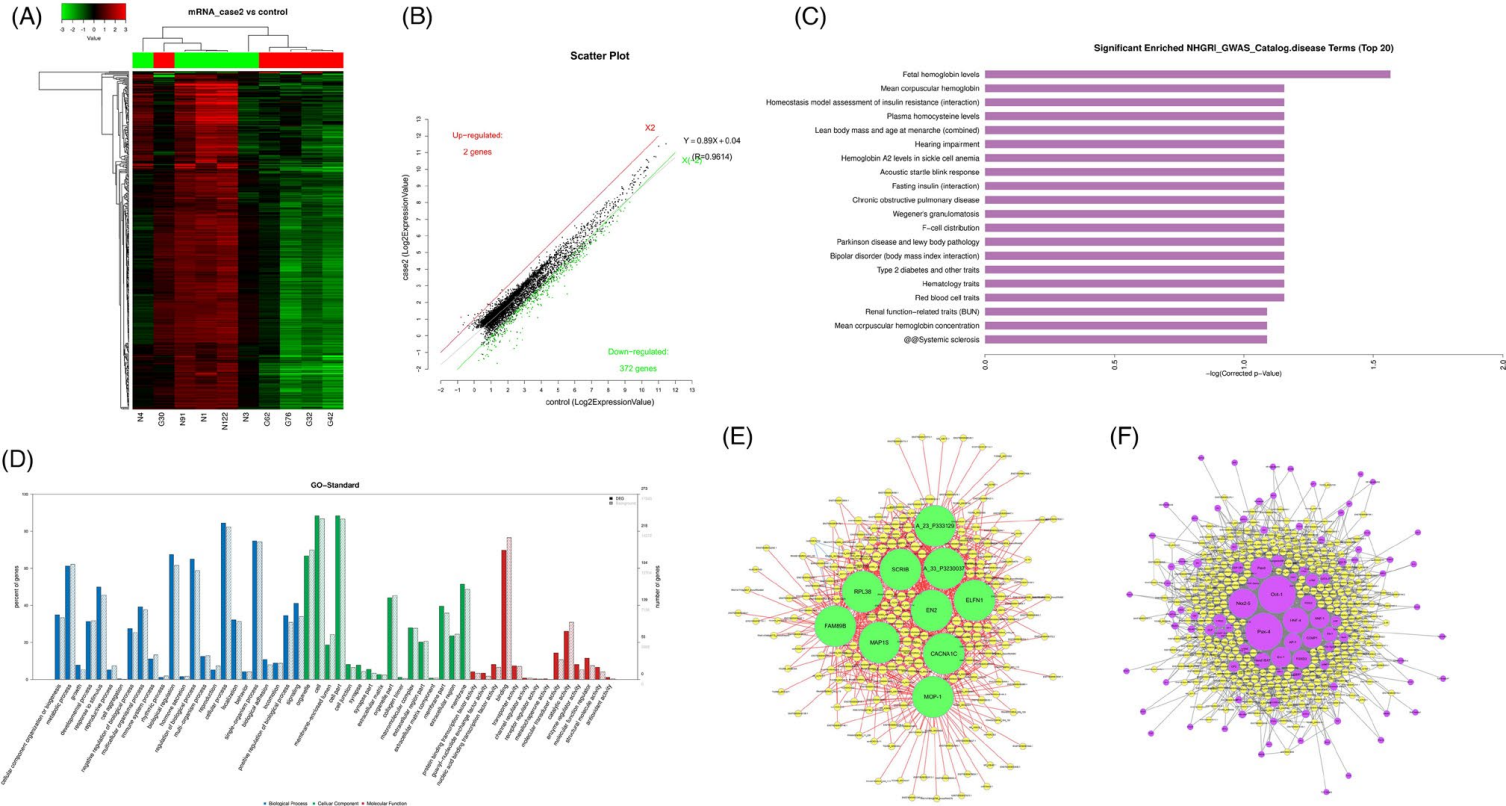
Profiling of mRNAs in the plasmas from gastric carcinoma (GC) patients with advanced TNM stages and normal controls. (A) Heat map shows the up‐regulated and down‐regulated mRNAs in case 2 vs. control group. (B) Scatter plot shows the up‐regulated and down‐regulated mRNAs in case 2 vs. control group. (C) Disease pathway analysis of mRNAs in case 2 vs. control group. (D) GO analysis of mRNAs in case 1 vs. control group. (E) Co‐expression analysis and cytoscape software were used to perform the two‐by‐two correlation calculation and hypothesis verification of mRNAs and non‐coding RNAs. (F) Cis‐prediction was performed to predict the association between mRNA and non‐coding RNAs according to their locations

**FIGURE 3:**
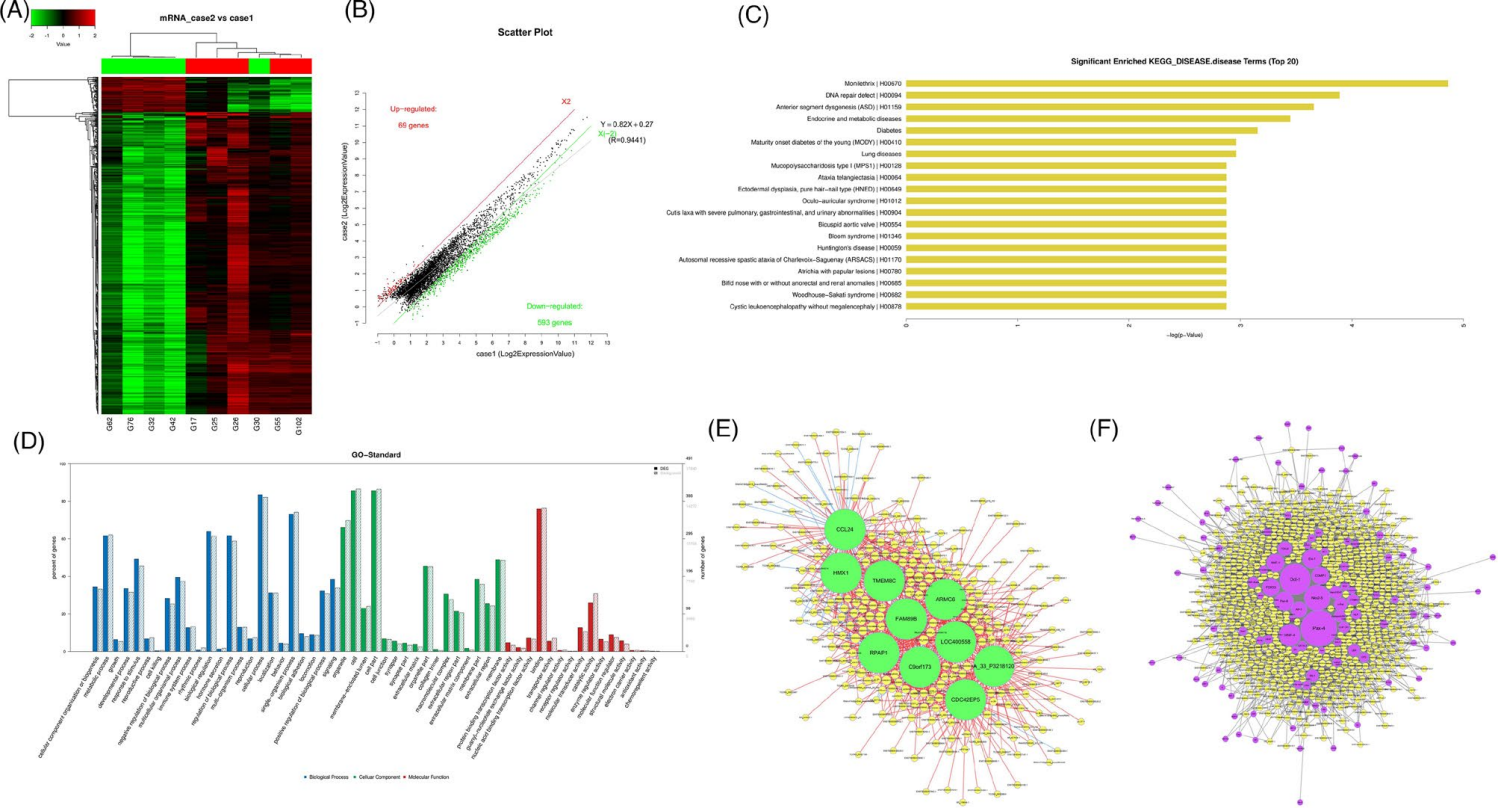
Profiling of mRNAs in the plasmas from gastric carcinoma (GC) patients in case 2 vs. case 1 group. (A) Heat map shows the up‐regulated and down‐regulated mRNAs in case 2 vs. case 1 group. (B) Scatter plot shows the up‐regulated and down‐regulated mRNAs in case 1 vs. control group. (C) Disease pathway analysis of mRNAs in case 2 vs. case 1 group. (D) GO analysis of mRNAs in case 1 vs. control group. (E) Co‐expression analysis and cytoscape software were used to perform the two‐by‐two correlation calculation and hypothesis verification of mRNAs and non‐coding RNAs. (F) Cis‐prediction was performed to predict the association between mRNA and non‐coding RNAs according to their locations

High‐throughput human mRNA microarray was conducted using plasma samples from 10 GC cases, including 5 cases with T3N0M0 (case 1) and the other 5 cases with T3N1‐3M0 (case 2), and 5 normal controls (control) to assess the differences of mRNA expression profiles in GC progression. Heat map and scatter plot were constructed to show differentially expressed mRNAs (*p* < 0.05 and fold change >2) in three groups: case 1 vs. normal group, case 2 vs. normal group, case 2 vs. case 1 group, respectively (Figures 1A,B, 2A,B, and 3A,B). The top 10 differently regulated mRNAs in each group were shown in Tables 1, 2, 3. Disease and GO analyses suggested that the mentioned differentially expressed mRNAs were relevant to cellular components, molecular functions, critical signaling channels, and several common diseases (Figures 1C,D, 2C,D, and 3C,D). Co‐expression analysis and cytoscape software were used to perform the two‐by‐two correlation calculation and hypothesis verification of mRNAs and non‐coding RNAs. Cis‐prediction was performed to predict the association between mRNA and non‐coding RNAs according to their locations (Figures 1E,F, 2E,F, and 3E,F).

**TABLE 1:** Top 10 differently expressed genes (case 1 vs. normal)

Gene name	Expression	*p* Value	Fold change	
ZNF865	Up	0.016414991	2.834144418	
LOC644285	Up	0.034627081	2.079781128	
HTRA2	Up	0.00721869	2.256966127	
CTSF	Up	0.000297996	2.232568217	
C18orf63	Down	0.010920353	12.16243441	
PHLDB2	Down	0.00739989	10.97625667	
PACRGL	Down	0.014980364	8.39960075	
OTUD3	Down	0.045650081	5.713357941	
KDM6B	Down	0.009375424	3.885350618	
ERLIN2	Down	0.012777738	3.647208263

**TABLE 2:** Top 10 differently expressed genes (case 2 vs. normal)

Gene name	Expression	*p* Value	Fold change
ORAI3	Up	0.023761631	2.152463478
HBA2	Down	0.001775119	8.846360662
TTC16	Down	0.009673159	8.629686421
GALR3	Down	0.002031316	8.568654553
LOC388210	Down	0.004375733	8.140500982
VSX1	Down	6.8256E−05	8.081846826
HBA2	Down	0.005427704	6.702367442
TMEM88B	Down	0.001525012	6.466931473
TSNARE1	Down	0.01476758	5.907858262
TAF15	Down	0.019838368	5.395599017

**TABLE 3:** Top 10 differently expressed genes (case 2 vs. case 1)

Gene name	Expression	*p* Value	Fold change
KDM6B	Up	0.011620134	4.329739975
GALR3	Down	0.013136168	4.939531173
HCN2	Down	0.004460222	4.290122895
SPSB4	Down	0.007653572	4.240747168
HECTD4	Down	0.006548522	4.119384333
ANK2	Down	0.006053924	4.083442118
DUSP15	Down	0.030112202	4.013029925
CHN2	Down	0.00968959	4.007987112
HR	Down	0.019184387	3.963257646
MEF2D	Down	0.014713692	3.925034532

### 3.2 HTRA2 is over‐expressed in GC and has remarkable clinical significance

**FIGURE 4:**
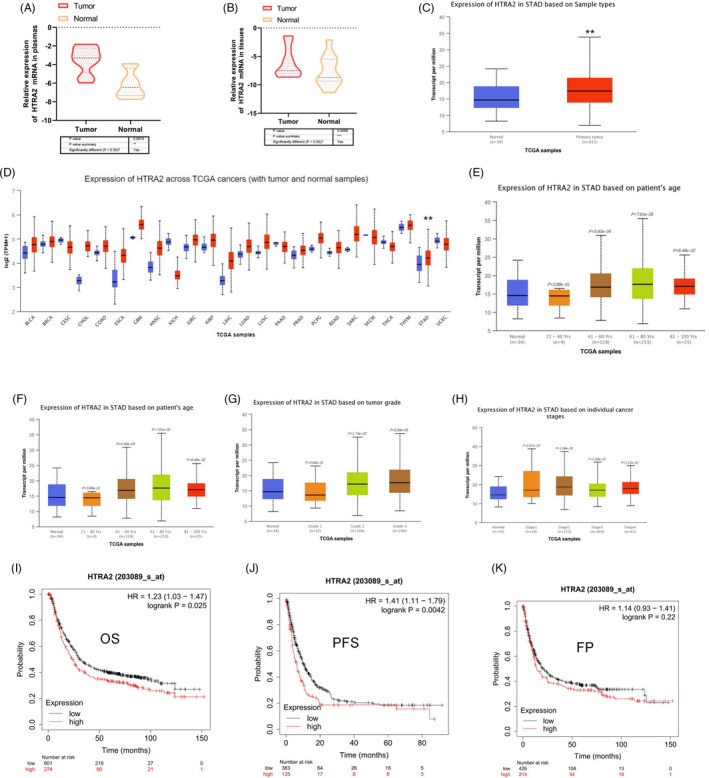
Clinical role of HTRA2 in gastric carcinoma (GC). (A) HTRA2 expression in GC tissues and normal tissues by qRT‐PCR. (B) HTRA2 expression in GC plasmas and normal cases by qRT‐PCR. (C) HTRA2 expression in GC tissues and normal tissues through TCGA database. (D) The expression level of HTRA2 mRNA in different type cancer tissues compared to normal tissue. (E‐H) The correlation between HTRA2 mRNA expression and age, tumor stage, tumor grade, and stages was analyzed. The Wilcoxon rank sum test was used to assess the significance of observed differences. (I‐K) The relationship between HTRA2 expression and GC patients OS, PFS, and FP. **p < 0.01.

According to stage‐related abnormal expression of mRNAs, we picked out one mRNA named HTRA2. Using qRT‐PCR, we have detected HTRA2 expressing state in GC plasma samples and healthy controls. The results indicated HTRA2 was significantly over‐expressed in GC plasma in contrast with control cases (Figure 4A). To confirm HTRA2 was derived from GC tumor, we further detected in primary cancerous and neighboring noncancerous tissues from GC cases. Results showed HTRA2 expressing states were significantly higher than that of neighboring noncancerous tissues (Figure 4B). The TCGA database shows results consistent with our conclusions (Figure 4C). The expression level of HTRA2 mRNA in different types of cancer tissues indicated that the expression level of HTRA2 in GC tissues was also higher than that in control group (Figure 4D). Subgroup analysis based on GC subtypes showed that HTRA2 mRNA expression was linked with age, grade, and individual cancer stages (Figure 4E‐H). To delve into the prognostic potential of HTRA2 in GC, Kaplan‐Meier Plotter was used. As presented in Figure 4I‐J, that higher HTRA2 expressing state displayed a correlation to shorter OS and PFS in contrast with lower expression but not FP.

### 3.3 HTRA2 plays a promoting role in GC cells in vitro

**FIGURE 5:**
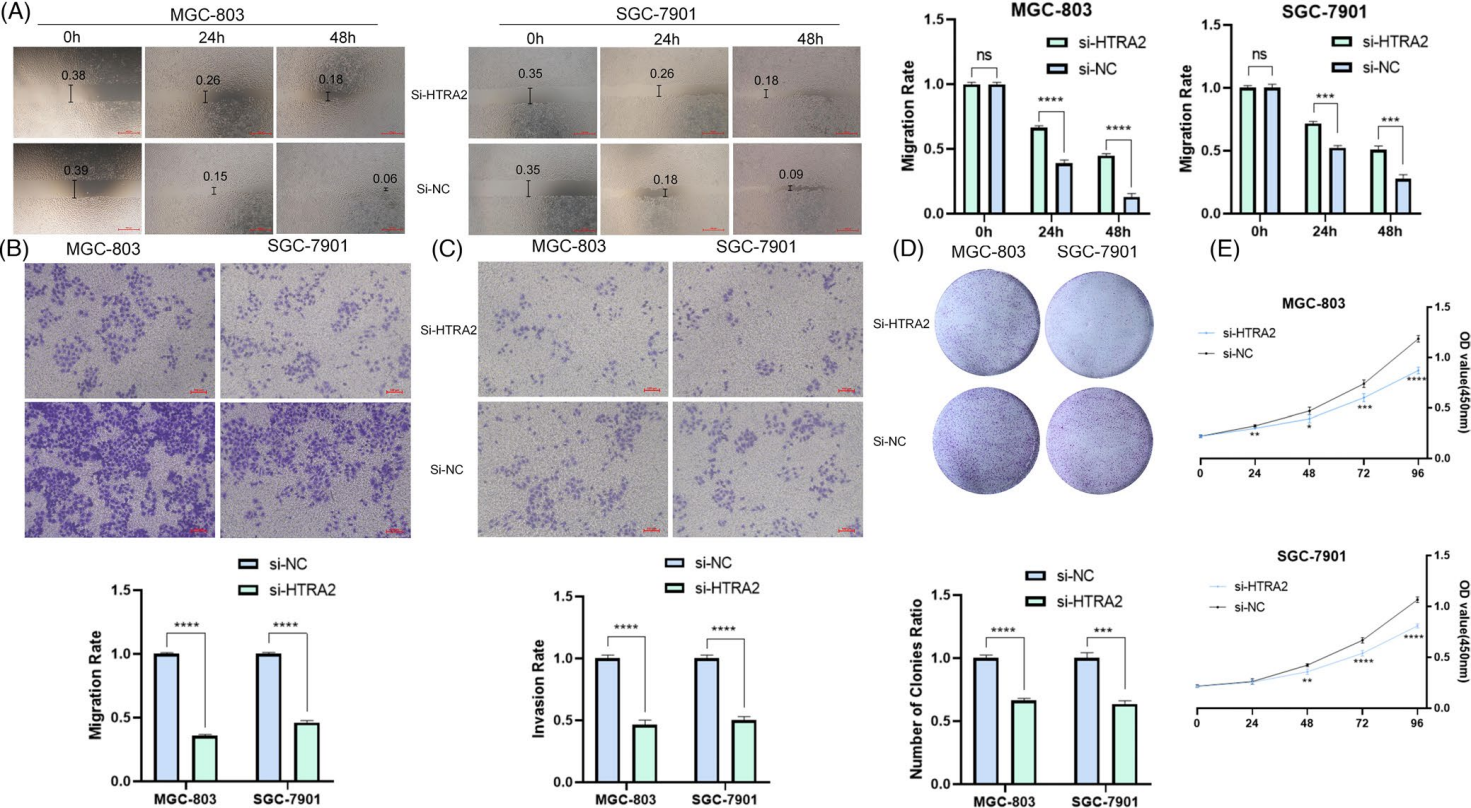
Knockdown of HTRA2 could inhibit the development of gastric carcinoma (GC) cell. (A) Scratch assay of knockdown of HTRA2. (B) Knockdown of HTRA2 could inhibit the migration of GC cell. (C) Knockdown of HTRA2 could inhibit the invasion of GC cell. (D) Plate cloning experiment of knockdown of HTRA2. (E) CCK8 results showed that suppression of HTRA2 by si‐HTRA2 exhibited a slower proliferation. *p < 0.05; **p < 0.01; ***p < 0.001; ****p < 0.0001

**FIGURE 6:**
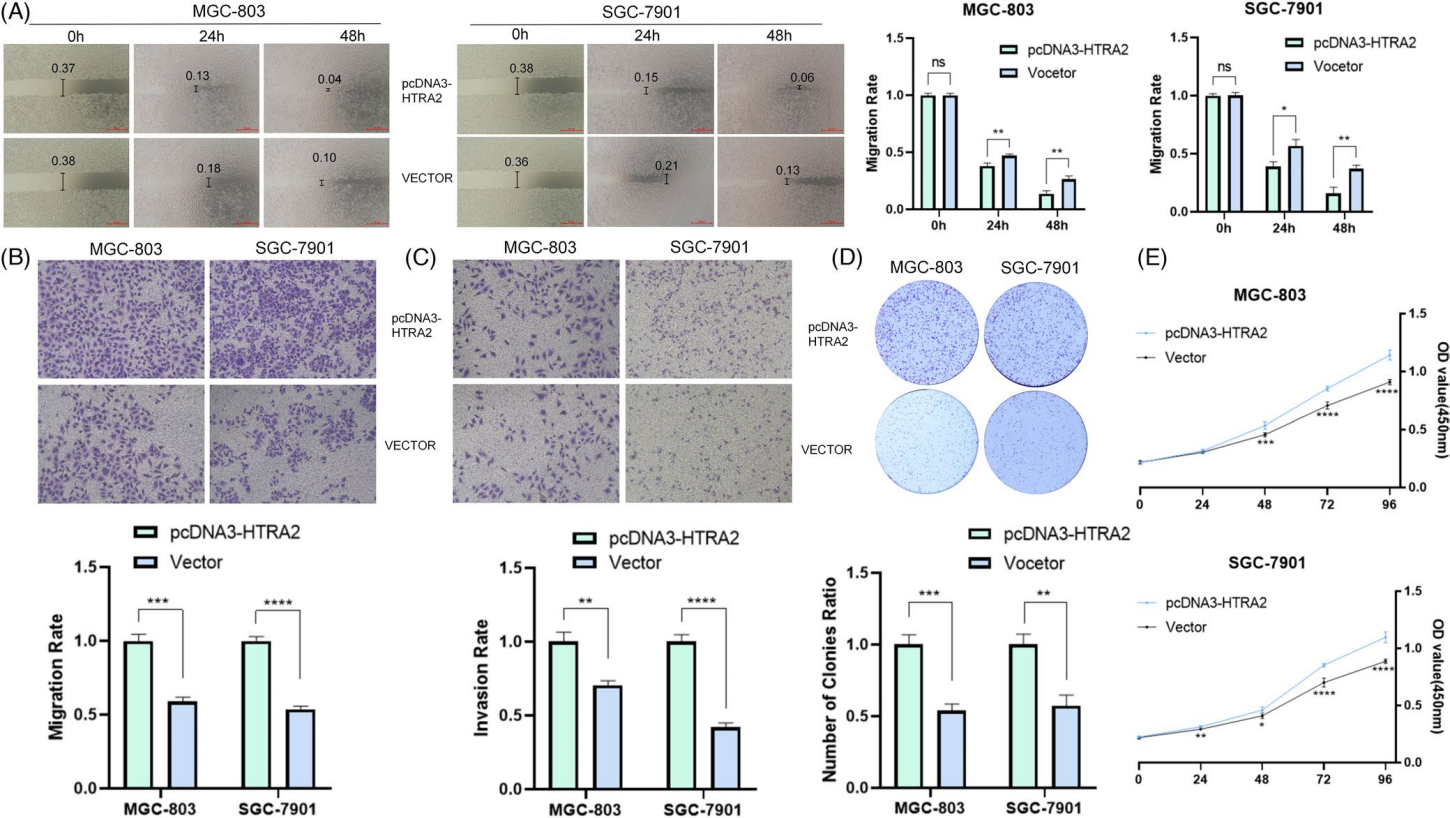
Over‐expression of HTRA2 could promote the development of gastric carcinoma (GC) cell. (A) Scratch assay of over‐expression of HTRA2. (B) Over‐expression of HTRA2 could promote the migration of GC cell. (C) Over‐expression of HTRA2 could promote the invasion of GC cell. (D) Plate cloning experiment of over‐expression of HTRA2. (E) CCK8 results showed that over‐expression of HTRA2 exhibited a higher proliferation.*p < 0.05; **p < 0.01; ***p < 0.001; ****p < 0.0001

To clarify the physiology‐related characteristic effect exerted by HTRA2 in tumors, HTRA2 expressing state received the effective knockdown or over‐expressing state inside GC cells. The results of a scratch‐wound assay demonstrated that suppression of HTRA2 exhibited a notably lower scratch closure rate than identified in controls in GC cell lines (Figure 5A). The HTRA2 suppression by si‐HTRA2 showed a decreased relative migrating and invading ratio (Figure 5B,C) as opposed to control groups according to transwell testing process inside the confluent monolayer of the GC cell lines under the culturing process. Plate cloning experiment and CCK‐8 assays showed that HTRA2 knocking down noticeably inhibited the proliferation of MGC‐803 and SGC‐7901 cell lines in contrast with control group (Figure 5D,E). Over‐expression of HTRA2 plays the opposite effect (Figure 6A‐E). The mentioned findings demonstrated that inhibition of HTRA2 can slow the progression of GC in vitro covering proliferation, invasion, and migration.

### 3.4 Genes and proteins co‐interacted with HTRA2 are associated with cellular response and metabolic signaling pathway

**FIGURE 7:**
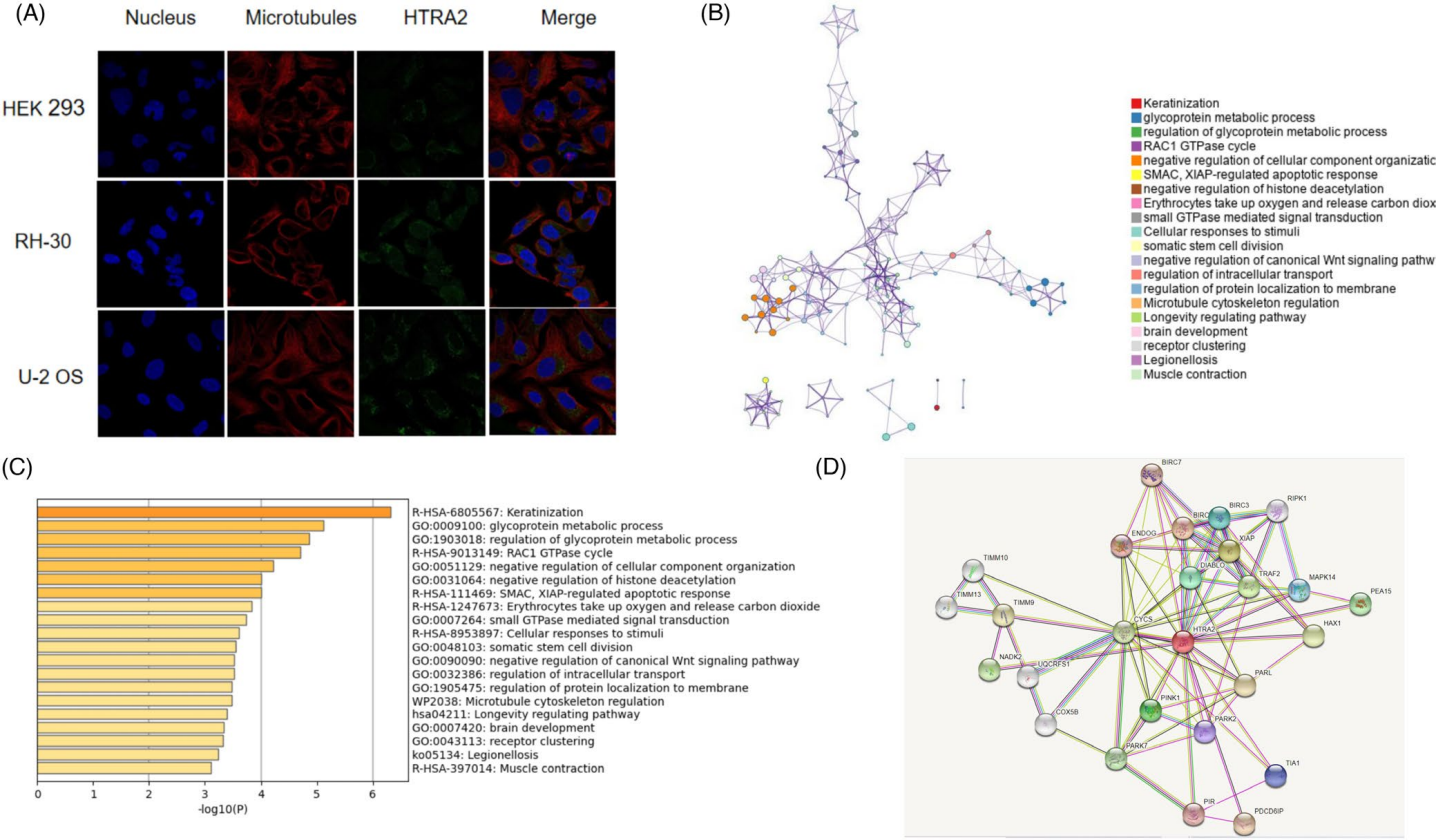
Genes and proteins co‐interacted with HTRA2 are associated with cellular response and metabolic signaling pathway. (A) HTRA2 location in cells. (B‐C) Pathway analysis of HTRA2 enrichment. The network of enriched terms colored by cluster ID; nodes that share the same cluster ID are typically close to each other. (D) A STRING interactive network interactions between HTRA2 and other proteins

According to the human protein atlas database, HTRA2 is located in the mitochondria of HEK293, RH‐30, and U‐2 OS cells (Figure 7A). Enrichment analysis of co‐expression genes performed using Metascape indicated that HTRA2 was primarily involved cellular response and metabolic signaling pathway (Figure 7B,C). A STRING interactive network was used to identify proteins like PINK1, PARL, and CYCS which can interact with HTRA2 (Figure 7D).

### 3.5 Research results of HTRA2 at single‐cell level

**FIGURE 8:**
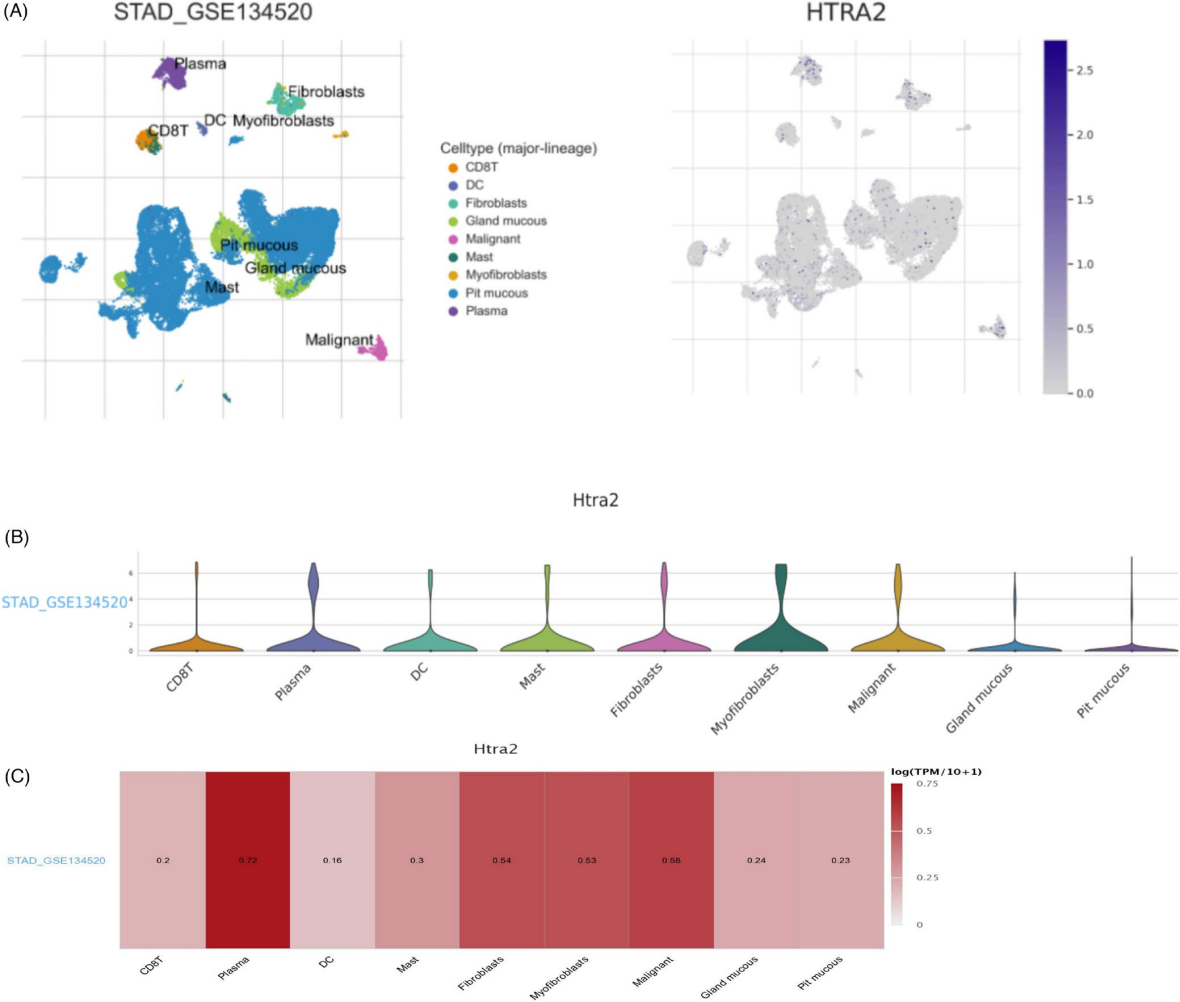
Research results of HTRA2 at single‐cell level. (A) Single‐cell cluster map of HTRA2. (B) Violin diagram displays the distribution of HTRA2 expression in different cells. (C) Heat map displays the value of HTRA2 expression in different cells

We studied the expression of HTRA2 at the single‐cell level. Single‐cell cluster map of HTRA2 in STAD GES134520 databases showed the distribution of HTRA2 expression in different cell clusters (Figure 8A). Violin and heat maps showed the specific expression of HTRA2 in different cells, which indicated that the HTRA2 mainly expressed at CD8T, plasma, DC, mast, fibroblasts, and malignant cell clusters (Figure 8B,C). These results suggest that HTRA2 may also function in stromal cells or immune cells other than cancer cells.

### 3.6 HTRA2 expression was correlated with immune factors

**FIGURE 9:**
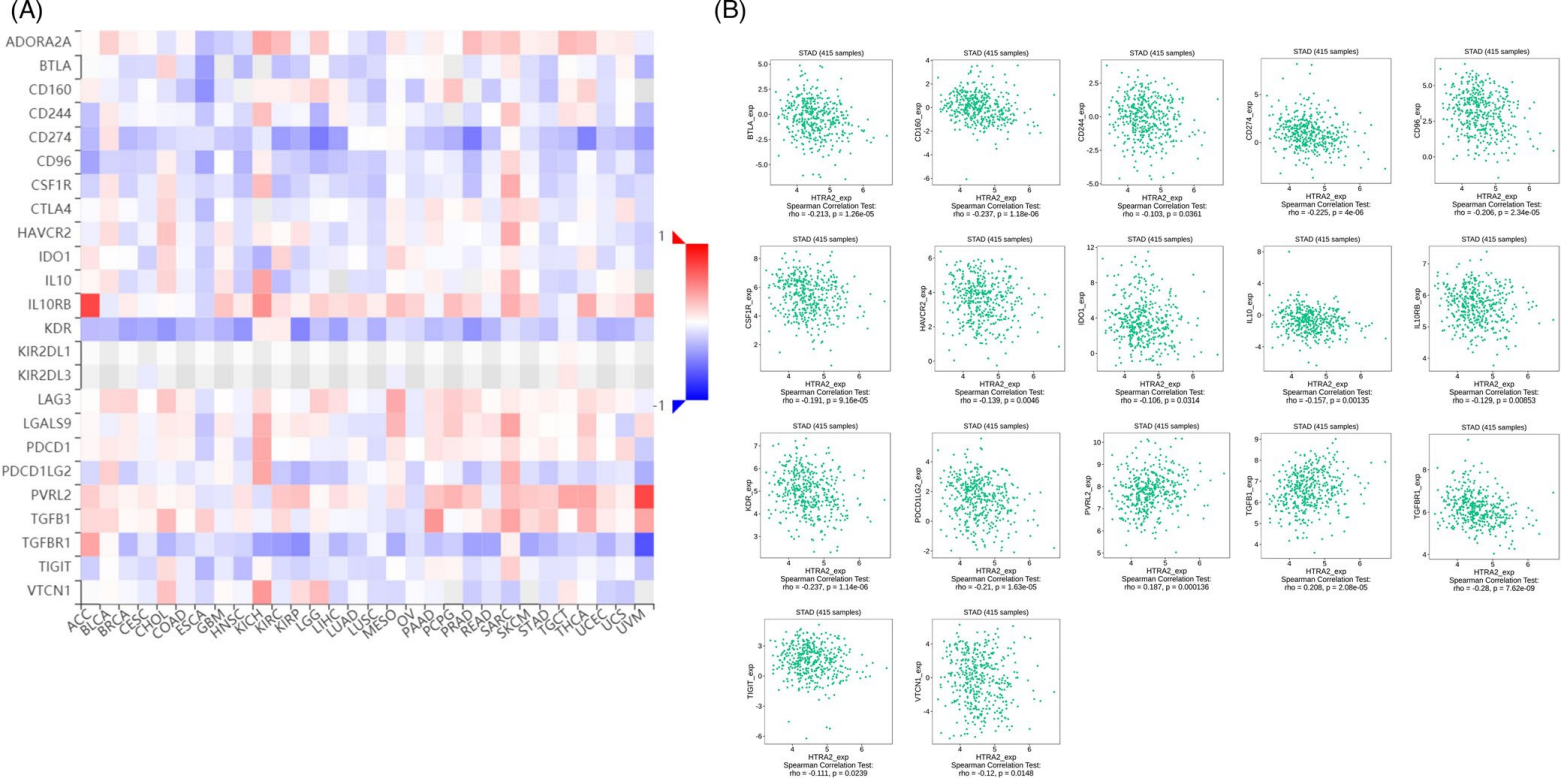
Correlation between HTRA2 expression and immunoinhibitors in gastric carcinoma (GC). (A) The heat map shows the correlation between HTRA2 and immunosuppressive factors in different cancers. (B) Line graph shows the correlation of HTRA2 with specific immune indicators in GC

**FIGURE 10:**
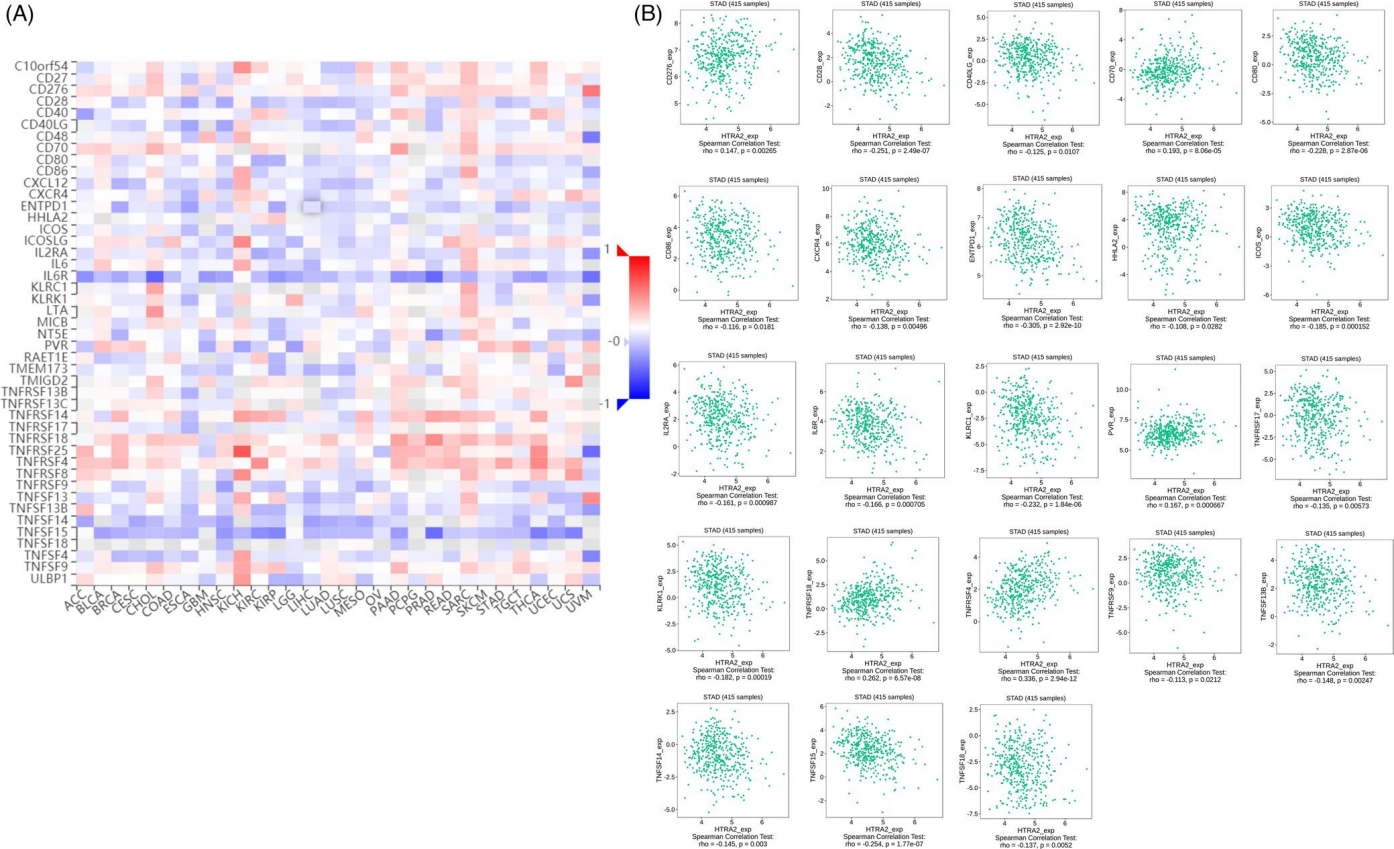
Correlation between HTRA2 expression and immunostimulators in gastric carcinoma (GC). (A) The heat map shows the correlation between HTRA2 and immunostimulators factors in different cancers. (B) Line graph shows the correlation of HTRA2 with specific immune indicators in GC

**FIGURE 11:**
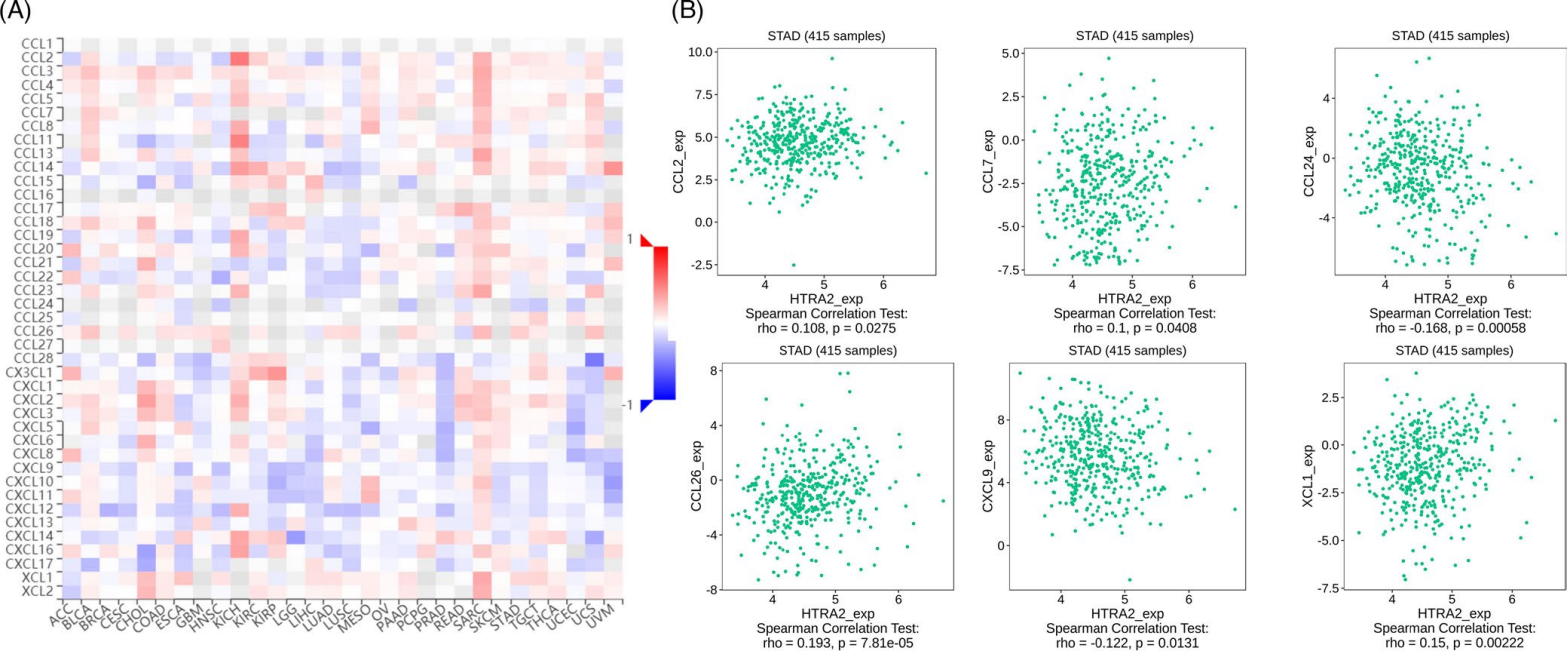
Correlation between HTRA2 expression and chemokines in gastric carcinoma (GC). (A) The heat map shows the correlation between HTRA2 and chemokines in different cancers. (B) Line graph shows the correlation of HTRA2 with specific immune indicators in GC

Existing studies have confirmed that the immune system is closely related to the occurrence and development of tumors. Therefore, we studied the relationship between the expression of HTRA2 and immune factors. As shown in Figures 9, 10, 11, there was a strong correlation between the expression of immunoinhibitors (CD274, IDO1, TIGIT, etc.), immunostimulators (CD80, CD86, ICOS, etc.), and chemokines (CCL2, CCL7, CXCL9, etc.) and the expression of HTRA2.

## 4 DISCUSSION

Gastric carcinoma refers to one of the most prevalent causes of carcinoma‐related death in the world. Cases usually diagnosed with advanced carcinoma due to the lack of noticeable symptoms. Hence, the development of GC screening tool would effectively reduce the overall mortality, and novel non‐invasive biomarking elements with better sensitivities and specificities are urgently needed.

Circulating cell‐free mRNA was first demonstrated in the 1990s in the plasma of cases with nasopharyngeal carcinoma.^8^ The discovery of circulating mRNAs in carcinoma cases holds great promise for the use of mRNAs as distinctive, non‐invasive carcinoma biomarking elements and therapeutic targets. Kopreski MS et al.^9^ reported that 5T4 mRNA was reproducibly detected in 42% of carcinoma patient sera, including breast carcinoma patient sera and lung carcinoma patient sera, but in only 12% of normal control. The presence of 5T4 mRNA in plasma and serum affords the opportunity to diagnose or stratify cases with carcinoma when tissue is not readily available. Lu et al.^10^ showed over‐expression of CTEN is associated with acquired gefitinib resistance in non‐small cell lung carcinoma (NSCLC). The plasma mRNA expression of CTEN was notably elevated in cases with NSCLC with acquired gefitinib resistance. Li et al.^11^ found a substantial fraction of intact mRNAs in human blood from healthy individuals and carcinoma cases and several of them could serve as biomarker for hepatocellular carcinoma (HCC) diagnosis with high diagnosis‐related efficiency. However, there were limited researches on the identification of circulating mRNA for the detection of GC.

HTRA2, located on chromosome 2p12, refers to an apoptosis factor inside the mitochondrial intermembrane area.^12^ Previous studies showed HTRA2 displayed a correlation to the development of various carcinoma, such as bladder transitional cell carcinoma and colon carcinoma.^13^, ^14^ Here, we reported tumor‐related HTRA2 mRNA's expressing state in the plasma of cases with GC and healthy ones, and its level was much higher in the serum of GC. Moreover, HTRA2 expressing state in the tissues of GC was found to be up‐regulated, which were consistent with the results of blood. The correlation with clinical factors of cases with GC was also analyzed, which showed that the high expression of HTRA2 displayed noticeably correlations to poor prognosis. For the in‐depth analysis of the correlation of HTRA2 expressing state to GC developing process, experiments were carried in vitro. In our study, we have observed that over‐expression of HTRA2 significantly promote cell proliferation while the knocking down of HTRA2 decrease cell variability in gastric carcinoma cell lines. Colony formation outcomes confirmed that HTRA2 enhance the ability of adhesion‐independent cell proliferation of GC. This indicated that HTRA2 may contribute to tumorigenesis of GC, probably due the unbalance between proliferation and apoptosis. Tumor invasion and metastasis are the major causes of morbidity and death for gastric carcinoma cases.^15^, ^16^ The present study has shown that HTRA2 over‐expression could increase the invasion and migration rate of both SGC7901 and MGC 803 cells. On the other hand, the suppression of HTRA2 by si‐HTRA2 exhibited an inhibitory effect on cell migration and invasion. The mentioned could partly explain that cases with elevated HTRA2 tend to have a poor prognosis. Nowadays, researches focus on the effect exerted by HTRA2 in GC are limited, and further extensive preclinical and clinical experiments are warrant to evaluate the effect and mechanism of HTRA2 before its potential application clinically.

Enrichment analysis of co‐expression genes performed using Metascape indicated that HTRA2 was primarily involved cellular response and metabolic signaling pathway. Liu Dan et al. reported that Omi/HTRA2 over‐expression in the elderly was negatively correlated with left ventricular function after exercise overload and positively correlated with myocardial Caspase‐9 apoptosis. Chromatin immunoprecipitation (ChIP) of aging hearts and plasmid transfection/RNA interference of H9C2 cells revealed that enhancement of heat shock factor 1 (HSF1) expression promotes Omi/HTRA2 expression by inducing the promoter activity of Omi/HTRA2 while also increasing mitochondrial apoptosis by up‐regulating Omi/HTRA2 expression.^17^ Liu Xin et al. found that mitochondrial Omi/HTRA2 promotes caspase activation through cleavage of HAX‐1 in aging heart.^18^ The basis of these studies has all verified one of our results, that is, HTRA2 is located in the mitochondria of cells.

A STRING interactive network was used to identify proteins like PINK1, PARL which can interact with HTRA2. Tain L S et al. described the characterization of mutations in Drosophila HTRA2, and genetic analysis of its function with PINK1 and parkin. Interestingly, they found HTRA2 appears to be dispensable for developmental or stress‐induced apoptosis. In addition, they found HTRA2 mutants share some phenotypic similarities with parkin and PINK1 mutants, suggesting that it may function in maintaining mitochondrial integrity.^19^ HTRA2 is phosphorylated on activation of the p38 pathway, occurring in a PINK1‐dependent manner at a residue adjacent to a position found mutated in patients with Parkinson's disease. HTRA2 phosphorylation is decreased in brains of patients with Parkinson's disease carrying mutations in PINK1.^20^ Yoshioka Hideyuki et al. showed that downregulation of PARL after ischemia is a key step in ischemic neuronal injury and that it decreases HTRA2 processing and increases neuronal vulnerability. In addition, processed HTRA2 released into the cytosol after ischemia contributes to neuronal injury via inhibition of XIAP.^21^ The interactions between HTRA2 and other proteins are not described here one by one, and more research reports are expected in the future.

Existing studies have confirmed that the immune system is closely related to the occurrence and development of tumors. Therefore, we studied the relationship between the expression of HTRA2 and immune factors. There was a strong correlation between the expression of immunoinhibitors (CD274, IDO1, TIGIT, etc.), immunostimulators (CD80, CD86, ICOS, etc.), and chemokines (CCL2, CCL7, CXCL9, etc.) and the expression of HTRA2. Hu Qingting et al. reported that CCR2 deficiency in breast cancer cell inhibited CCL2‐mediated growth and invasion, corresponding to decreased ALDH1A1 expression and increased HTRA2 expression. ALDH1A1 and HTRA2 expression was modulated in CCR2‐deficient and CCR2‐over‐expressing cell lines. They found that ALDH1A1 and HTRA2 regulates CCR2‐mediated breast cancer cell growth and cellular invasion in a CCL2/CCR2 context‐dependent manner.^22^ We also studied the expression of HTRA2 at the single‐cell level and found that HTRA2 mainly expressed at CD8 T, plasma, DC, mast, fibroblasts, and malignant cell clusters. These results suggest that HTRA2 may also function in stromal cells or immune cells other than cancer cells. However, at present, there are few studies on the correlation between HTRA2 and cancer in immune cells or stromal cells. It is expected that more studies will make breakthroughs in these aspects in the future.

In conclusion, we applied a systematic method to analyze several mRNAs capable of distinguishing cases with GC. We found HTRA2 was over‐expressed in GC plasma in contrast with control cases. According to Kaplan‐Meier overall surviving curve, cases carrying higher HTRA2 expression achieved decreased total surviving time. Though the outcomes here are rudimentary, this study underpins the application of the mentioned mRNAs to clinical practice. The particular regulatory effect exerted by them still needs to be further explored for clinical application.

## CONFLICT OF INTEREST

The authors declare that they have no conflicts of interest.

## Data Availability

Data sharing is applicable.
